# Actual data on epidemiological evolution and prevention endeavours regarding traumatic brain injury

**Published:** 2015

**Authors:** C Popescu, A Anghelescu, C Daia, G Onose

**Affiliations:** * Physical (neural–muscular) and Rehabilitation Medicine Clinic Division, “Bagdasar-Arseni” Teaching Emergency Hospital, Bucharest, Romania; ** “Carol Davila” University of Medicine and Pharmacy, Bucharest, Romania

**Keywords:** traumatic brain injury, epidemiology, prevention

## Abstract

**Background:** Knowledge of the epidemiology of traumatic brain injury (TBI) is required both to prevent this disorder and to develop effective care and rehabilitation approaches for patients.

**Objective:** The aim of this article is to find solutions to decrease the incidence of TBI and offer recommendations for their prevention.

**Material and methods:** We analyzed epidemiological studies on TBI by performing a systematic review of literature, using information reported by different centers, collecting data on demographics, showing characteristics of TBI including incidence, identification of risk groups on differences in age, gender, geographical variation, severity and mortality.

**Results:** Studies suggest that the incidence of TBI is between 18 and 250 per 100,000 persons per year. Men and people living in social and economical deprived areas, usually young adults and the elderly are high-risk groups for TBI.

**Discussion:** Prevention remains the “key point” in medicine and especially for TBI, saving the patient from unnecessary often-harsh sufferance.

**Conclusions:** Most public epidemiological data showed that TBI is a major cause of mortality and disability. The effort to understand TBI and the available strategies to treat this lesion, in order to improve clinical outcomes after TBI, may be based on an increase in research on the epidemiology of TBI. A coordinated strategy to evaluate this public health problem in Romania would first of all rely on a related advanced monitoring system, to provide precise information about the epidemiology, clinical and paraclinical data, but concerning the social and economic connected consequences, too.

**Abbreviations:** CNS = central nervous system, ED = emergency department, EU = European Union, FTE = Full Time Employees, GCS = Glasgow Coma Scale, TBI = traumatic brain injury, US = United States, WHO = World Health Organization.

## Background

This article approaches a number of aspects related to the epidemiology of traumatic brain injury (TBI) as it became a worldwide public health problem, which can lead to long-term disability, which presents by far consequences that should not be neglected, with personal “destiny fractures” and substantial socio-economic costs for the affected people and their relatives, but also for the community. Therefore, there is a great need for more effective ways to prevent TBI. 

Knowledge about the epidemiology of TBI is necessary in order to prevent this disorder, to improve public health programs and to efficiently implement protocols of managing it, being able to provide effective medical care and rehabilitation services for patients, in view of obtaining better outcomes and reducing not only physical disability but also cognitive and emotional sequels that tend to evolve in relation to the severity of trauma [**1**,**2**]. Therefore, patients with consequent disabilities, required for treatment and rehabilitation, should always be in the center of extended and integrated, coherent approaches, in order to eventually prevent long term medical and psychological complications as well as the socio-professional, family and/ or economic effects.

## Methods

Analyzing the epidemiological studies on TBI during an extended period, we found that while collecting data, several problems could occur, including the fact that many patients with moderate TBI do not present to the hospital and those who do, can be discharged from the emergency units without appropriate documentation. Additionally, deaths following severe TBI that result after accidents or during transport to the hospital cannot be fully accounted for collecting data as necessary in related epidemiological studies [**3**]. Other confusing variables in determining the epidemiology of TBI include the use of different definitions that may not clearly characterize the type of lesion, as well as the appearance of conflicting data within the results of diagnostic imaging used at separate times [**3**].

The pressing need to advance research in TBI is obvious, especially when considering the predictions of the World Health Organization (WHO) that TBI will become the third leading cause of death and disability in the world, by 2020 [**4**,**5**].

TBI is the leading cause of death in the first forty years of life, and the number of years lost due to accidents is far greater from cardiovascular or neoplastic disease [**6**].

The first place in the etiological structure of acute brain lesions is held by TBI, followed by ischemic or hemorrhagic stroke, brain tumors, diffuse cerebral hypoxia after resuscitation [**7**]. 

**Incidence**

The general incidence of TBI in developed countries is frequently stated to be 200 of 100.000 population/ year, including only patients admitted to hospitals [**8**]. 

**Fig. 1:**
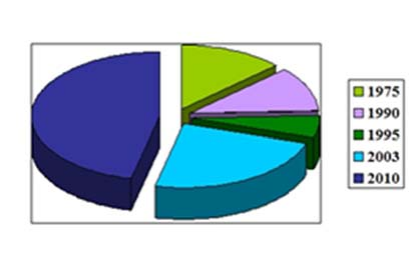
Graphical representation of the annual incidence of TBI in US (synthesis based on data from [**4**,**9**-**12**])

We analyzed the data from several epidemiological studies and based on this, we compared the incidence of TBI for different periods of time (**Fig. 1**).

More precisely, the data analyzed reveal that the annual incidence rate of TBI hospitalizations in the United States (US) has been declining since 1975, when it peaked 234 per 100,000 population [**9**]. 

Later, in the 1990’s, the incidence of TBI in the US was estimated at 200/ 100,000 population, meaning 500,000 new cases in a population of 250 million inhabitants [**10**], while in 1995, the mean annual incidence rate of hospitalized survivors after TBI was 99 per 100.000 population (260,000 cases) [**9**].

More recently, in 2003, there were over 1 million emergency department (ED) presentations due to head trauma (423 of 100.000), thus resulting in an increase of them [**4**,**11**].

Within the last decade (2001–2010), rates of TBI-related ED visits increased by 70%. Therefore, in 2010, in the US approximately 2.5 million people sustained a TBI, representing an important increasing rate: to 823/ 100,000 [**12**]. 

TBI generates about 1 million presentations to the emergency units per year in the European Union (EU). About 235 per 100,000 inhabitants/ year are frequently hospitalized, the incidence including both inpatients and persons who died after TBI [**13**]. 

According to the preliminary results of an epidemiological research conducted in the neurosurgery departments in Romania, in 1997, by the group “Neurotrauma” of the Romanian Society of Neurosurgery, TBI incidence varied between 25-95%, being the leading cause of death [**14**]. 

**Incidence by age**

**Fig. 2:**
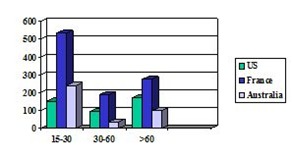
The graphical representation of comparative incidence of TBI in age groups in US, France and Australia (synthesis based on data from [**8**])

Comparing the incidence of TBI in adolescents/ young adults (15-30 years) age group, among different geographical areas, it has been concluded that in the US it reached values between 154 to 415 per 100,000, in France it achieved 535 per 100.000, and in Australia it attained 240 per 100.000 persons. While the incidence among adults in US was 93 per 100.000 persons, in Australia it reached 35 per 100.000, whereas in France it reached 190 of 100.000. Regarding the incidence in geriatric population, in US it was about 173 of 100.000, in France was nearly 275 of 100.000 and in Australia, it was 100 of 100.000 and (**Fig. 2**) [**8**]. 

**Incidence by gender**

Males are more likely to sustain a TBI than females, the ratio being 2/ 1 in their favor. This ratio is near in parity with the one by age, because of the increased risk of TBI caused by falls, which is similar in both sexes [**3**,**15**]. 

**Fig. 3:**
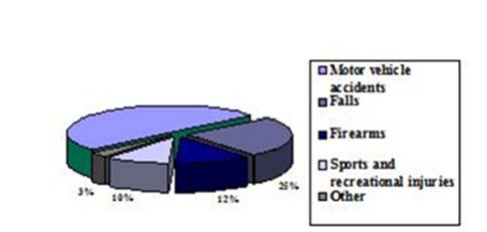
Graphical representation of incidence by mechanisms of production in TBI (synthesis based on data from [**3**,**8**])

**Incidence by causes and mechanism of production** (**Fig. 3**). 

Regarding the mechanism of injury in the US, motor vehicle accidents - the leading cause of TBI in the general population - were responsible for 50% of all TBI.

Falls - the second leading cause of TBI in the US - were accounting for about 20-30% of all TBIs. In elderly, over 65 years, falls are the most common causes of TBI [**3**]. The very young people, also frequently suffer TBIs due to falls [**3**]. Overall, findings suggest that the overall annual rates of fall-related TBI deaths increase dramatically with increasing age [**16**]. 

**Fig. 4:**
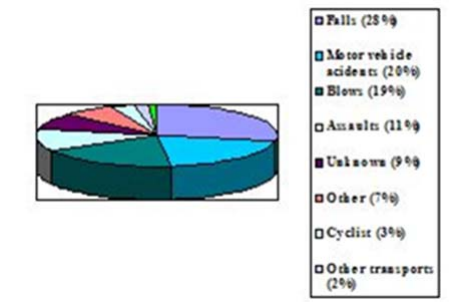
Graphical representation of the percentage amount of the TBI causes in the US (synthesis based on data from [**18**])

More accurately, the annual average percentage of the presentations in the emergency room, of the hospitalizations and deaths regarding the causes of injuries due to TBI in the US, between 1995 and 2001, were found in a study as it follows: 28% from falls, 20% by motor vehicle accidents, 19% by blows, 11% assaults, 9% unknown, 7% other, 3% no motor vehicle accidents (cyclist), 2% other transport, 1% suicide (**Fig. 4**) [**17**]. 

Firearms are the third leading cause of TBI (12% of all TBIs), and a leading cause of TBI among persons aged 25-34 years, being more prevalent in African Americans than in Caucasians [**3**].

Sports and recreational injuries were responsible for 10% of all TBI [**8**].

A Romanian study showed that during 2001 and 2005, 333 TBI patients, with the mean age around 40 years, needing rehabilitation, were transferred to Physical & Rehabilitation Medicine Clinic Division of “Bagdasar-Arseni” Clinical Emergency Hospital, Bucharest, the etiology of head trauma being the following: car accidents – 194 cases (58%), falls from height – 63 cases (19%), aggression - 44 cases (13%), other causes - 32 patients (10%) [**18**]. 

**Incidence by severity**

The Glasgow Coma Scale (GCS), described by Teasdale and Jennet in 1974, is the widespread used clinical classification for TBI severity, also having prognostic value [**8**], an exponential increase of mortality being correlated with the decrease in GCS score [**19**]. 

Classically, the GCS is stratified into 3 layers of severity by score frames, as it follows: severe (8 points or less), moderate (9-12p) or mild (13-15 p) [**20**].

Yet, a Romanian study asserted that a score over 7 shows a 90% probability of a favorable evolution or moderate neurological deficit. A score below 7 suggests a considerable increase in mortality or persistent vegetative state, the risk being 60-90% when GCS score is 3 [**19**]. 

Accordingly, about 80% of TBI are found to be mild, 10% moderate and 10% severe [**8**].

However, as described in another related epidemiological article, in the US, between 58% and 73% are mild, between 8% and 25% are moderate and between 6% and 8% are severe [**8**].

Approximately 1.74 million people suffer minor TBIs causing temporary disability for at least one day [**3**] Dawodu ST.

In the EU, the ratio between mild/ moderate/ severe TBI is 22/ 1.5/ 1 [**13**].

**Prevalence**

The prevalence in the US is estimated to be of around 5 million people, i.e. 1-2% of the population have a disability related to TBI, while in Europe, 7,7 million population surviving after brain trauma, suffer from disabilities [**21**]. 

Prevalence of TBI is not well documented very probably because most of the cases are not hospitalized [**3**], the survivors are rather difficult to be systematically followed up on long term.

Estimations based on existing disabilities, showed that 2.5-6,500,000 Americans are living with TBI sequels, which often need long term complex management and care. As a short related complementary digression, it should be noted that in the US, 2.8 is considered the necessary number of full time employees (FTE) for such a patient: most often for months, sometimes for life [**22**].

 The number of patients who require extended hospitalization and rehabilitation is a different measurement of the prevalence. In this respect, there are disparities between different parts of the world; these may be allocated to methods for checking various cases of TBI and in the way of referring to the cause of the lesion [**3**].

As prevalence refers to a long-term related status, precise data regarding TBI are helpful, including for improving rehabilitation programs [**23**]. 

**Mortality and morbidity**

**Fig. 5:**
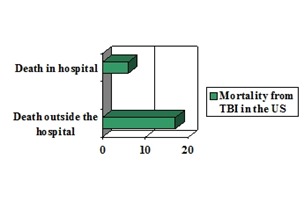
Graphical representation of comparison between mortality from TBI, in the US, among hospitalized and non-hospitalized patients (synthesis based on data from [**8**])

TBI is the major cause of death injury induced. In the US, TBI represents about 40% of all deaths due to acute injury and the leading cause of mortality among people younger than 45 years. To note that, mortality due to TBI in the US was between 10 and 35 per 100.000 persons but decreasing across most etiologies and that death occurring outside hospital was of about 17 per 100,000 people, compared to that of hospitalized patients, which was estimated at about 6 per 100,000 people (**Fig. 5**) [**8**]. 

In the EU, TBI mortality rate has an average of 15 new cases/ 100,000 inhabitants/ year [**13**], ranging from 9 in England [**10**] to 22 in France [**8**]. 

According to preliminary results of the epidemiological research conducted in Romania, in 1997 by the group “Neurotrauma” of the Romanian Society of Neurosurgery, mortality varied between 60-90% for severe TBI (much higher than the average of this indicator in the EU, which was around 31% in 1996) [**14**]. 

**Financial cost**

Aside from the human suffering, which is incalculable, the direct and indirect costs are enormous, being appreciated, in the US, as amounting to $9- $10 billion spent on acute TBI and rehabilitation services annually. TBI may cost an additional amount of $1 billion per year by lost wages and/ or income taxes, and increased public assistance [**24**]. These injuries have both short-term and long-term effects on individuals, their kin and society; therefore, their overall cost is huge.

## Discussion

Prevention remains essential in medicine and especially for TBI: exempts the patient from unnecessary suffering, requires less time and expense budget allocated to health. Until prophylaxis is given the necessary importance, accidents generally and those with central nervous system (CNS) involvement in particular, will continue to drain an important part of the health expenditure, efforts and sufferance.

To reduce the incidence of TBI and thus to improve mortality and disability prevention, should be a national and also an international priority.

The number of head injuries is increasing for several reasons: the development of the automotive industry, multiplying domestic and work accidents and respectively assaults. 

Globally, alcohol and/ or drugs intake are an important cause of death, also in relation to traffic accidents, so, a careful monitoring of the blood concentration in persons involved in road events and a good implementation of regulations regarding driving under the influence of alcohol and drugs must be rigorous [**3**]. In the context of many medicines that alter both the attention and promptness of reactions, persons in treatment, with potential risk may be repeatedly warned of the need for temporarily giving up driving.

The gravity of the medical and socio-economic consequences entailed by TBI and the fact that currently there is still no effective treatment once CNS lesions are formed, makes prevention a major element of the medical practice and not only. 

Also based on this overview endeavor, we reckon that an essential prevention tool is related literacy, which still seems not to be satisfactory. Most of the victims - not enough educated about TBI - are also individuals with a modest social condition, the material status being considered a risk factor for brain injury, suggesting a direct link between the social and the appearance of a TBI. Adopting a national educational program to prevent accidents should be mandatory in order to obtain a lower mortality and morbidity rate caused by TBI.

Prophylaxis through education should begin by developing effective strategies such as providing related knowledge starting from an early age and continued by suitable means throughout life, including with documentation on such preventable events and last but not least, by assuming legislation implementation.

Educational prophylaxis has a very strong conveyor through the mass media. A specific example in this respect is the campaign carried out, during 2006 and 2008, by the Romanian Public Television in partnership with the National Traffic Police Inspectorate, in the form of the broadcast: the “Show mirror" – including with a marked educational role, supported by presenting the medical consequences, sometimes devastating, of road accidents - systematically/ weekly emphasized by the Head of our Clinic Division, Prof. G. Onose and some distinguished colleagues.

Additionally, very recently (March 2015), our Clinical ward participated in the making of a short movie promoting preventive aspects on traffic accidents entitled “The Safety of Young Drivers. Our Responsibility”, produced by a television company in a related national campaign sustained by the Romanian Association for Safety and Health at Work (medical contributors to it: Onose G. and C. Daia).

An interesting article [**25**] refers to the protective role of a very well known accessory device to be warred by different types of bikers. More precisely, it determines a 50,2% decrease in motor vehicle–related deaths “over 18 years”, after the introduction of the mandatory helmet law in Taiwan (passed in June 1997). It must be noted that in Romania wearing a helmet on motorcycle has been mandatory for almost 40 years.

Consensually, the incidence of major causes of TBI has been observed to significantly decrease the following of the introduction of preventive safety measures: wearing a seat belt in a car, wearing a helmet on a bicycle, motorcycle and other open unrestrained vehicles, when participating in contact sports, skiing, snowboarding, skateboarding, providing adequate lighting, especially on stairs for people with poor vision and/ or adequate carpets or other coverings to prevent falls on slippery surfaces, for individuals with gate difficulties, placing bars on windows to prevent children from falling or respectively suicide attempts, animals (including/ mainly domestic) assaults. Such measures should constantly and rigorously be applied.

Developing a plan of prevention must be done by a team composed of people with backgrounds and different fields of expertise, and also by involving political, legislative and educational decisions/ measures that can support effective preventive programs. Therefore, although the use of other countries’ experience is valuable, each national preventive strategy should be adapted to the specific realities and needs of every state. Finally yet importantly, speaking about advanced achievements in this domain - to be seen in different developed zones around the word - having a national dedicated electronic registry is, in our opinion, another related valuable inner aim.

It should also be taken into account that an effective prevention of TBIs means not only the reduction of bio-psychological consequent sufferance but also mitigation of the treatment, cares and rehabilitation, individual/ kin and overall societal, related costs.

## Conclusions

According to the WHO and most public epidemiological data, lesions from TBI are a leading cause of death and respectively, for the survivors and their relatives, huge global burden below age 60 years [**26**] but not only, and also a significant multimodal challenge for the community: medically, socially, economically, educationally, advocationally and regulatory. 

Continuous research/ documentation on such primary preventable events, education by appropriate means and implementation of appropriate legislation remain basic necessary prophylactic measures.

**Conflict of interests **

The authors declare no conflict of interests.
